# The relationship of intimate partner violence on depression: the mediating role of perceived social support and the moderating role of the Big Five personality

**DOI:** 10.3389/fpubh.2024.1402378

**Published:** 2024-07-03

**Authors:** Zhenni Luo, Yan Guan, Yun Li, Weihong Xu, Lu Li, Siyuan Liu, Haozheng Zhou, Xuanhao Yin, Yibo Wu, Jiangyun Chen

**Affiliations:** ^1^School of Health Management, Guangzhou Medical University, Guangzhou, China; ^2^School of Public Health, Southern Medical University, Guangzhou, China; ^3^School of Public Health, Peking University, Beijing, China; ^4^Center for WHO Studies and Department of Health Management, School of Health Management of Southern Medical University, Guangzhou, China

**Keywords:** intimate partner violence, depression, perceived social support, five-factor personality model, resiliency

## Abstract

**Introduction:**

This study aimed to explore the influence of Intimate Partner Violence (IPV) on depression, the mediating role of social support, and the moderating role of the Big Five personality traits in the relationship between social support and depression.

**Methods:**

Participants were recruited from Mainland China, using a stratified random sampling and quota sampling method. From June to August 2022, a diverse group of 21,916 participants (ranging from 12 to 100 years old) completed the Intimate Partner Violence Scale, Patient Health Questionnaire, Perceived Social Support Scale, and Big Five Inventory-Short Version.

**Results:**

IPV was significantly positively correlated with depression and significantly negatively correlated with perceived social support. Perceived social support plays a mediating role in the link between IPV and depression.

**Discussion:**

Healthcare workers should assess social support and provide adequate care or recommendations for increasing social support when patients with IPV report depressive symptoms. Patients can be coached by professionals to improve their resiliency by developing or nurturing more optimistic personality traits.

## 1 Introduction

Intimate partner violence (IPV) is a serious, prevalent, and preventable public health problem that disproportionately affects women worldwide. Intimate partner violence describes physical or sexual assault, or psychological harm, of a spouse or sexual intimate (1). Its prevalence varies by social, economic, and cultural background, although it exists in all demographic groups (2). Globally, 35% of women have experienced intimate partner violence (3). The regions of Africa and Southeast Asia have reported the highest rates of IPV; higher-income regions like Europe and the Western Pacific exhibit a lower incidence of IPV, with reported prevalence rates ranging from 23 to 25% (4). Regarding different types of IPV, prevalence rates among Chinese college students were reported as 16.7% for physical harm, 18.9% for sexual coercion, and 51.8% for psychological harm (5). Beyond physical harm, these other forms are likely underreported.

Owing to its high occurrence, IPV is a significant concern for public health, greatly affecting both physical and mental well-being. Fractures, cuts, head injuries, sexually transmitted infections, unintended pregnancies, and pain disorders are some of the physical consequences that can occur from physical or sexual violence. Increasing evidence indicates that individuals who have encountered IPV are more susceptible to mental health issues (6), such as heightened vulnerability to depression, anxiety, post-traumatic stress disorder (PTSD), and suicide (7). However, there is less epidemiological evidence of relevance in contemporary Chinese populations. Exploring the relationship between IPV and depression under the background of China’s characteristic culture and system is helpful to enrich the relevant theoretical framework from different cultures and institutional groups.

### 1.1 IPV may relate to depression

For victims, apart from physical injuries, mental health problems especially depression and anxiety, are common (8). According to Bonomi et al. (9), women who experienced IPV within the previous year had a risk 3.26 times higher of developing depression compared to women who were not subjected to abuse. A positive association was found between IPV and postpartum depression, with IPV emerging as a primary predictor of postpartum depression compared with women who did not experience IPV (10). Depression in those experiencing IPV can lead to poor recovery from continuing abuse and an overall decrease in health for these individuals. Therefore, we predicted that IPV is associated with clinical depression in severe cases.

### 1.2 The mediating role of perceived social support

Individuals can feel valued, loved, and appreciated when they have social support, and these feelings allow them to believe that they are part of a community. Sources can include family members, friends, neighbors, and colleagues. This support may improve feelings of self-efficacy and enhance one’s ability to seek help (11), as well as reduce exposure to IPV. For example, talking to a family member at least once a month during pregnancy may decrease risk of both IPV overall and repeated episodes of IPV (12). This indicates that social support may protect against IPV.

Social support also mitigates the influence of IPV on subsequent mental health problems (13). Studies conducted on impoverished urban populations in the US indicate that individuals who encounter IPV are more likely to face mental health challenges if they are exposed to higher levels of violence and endure various forms of abuse, and social support may be an important protective factor for them (14). Adams et al. (15) also found that social support was an important factor in preventing depressive symptoms, and a lack of perceived social support could result in a higher prevalence of depression. Family support may be a particularly significant mediator in the relationship between abuse and mental health outcomes, including anxiety, depression, and PTSD (16). Moreover, seeking social support can improve individual psychological capacity and mitigate the negative mental health consequences of IPV, including depression (17, 18).

### 1.3 The moderating role of personality traits

The Big Five personality theory (also called the five-factor model) is commonly used in personality research. The Big Five Model represents five basic and universal traits related to broad personality dimensions: extraversion, agreeableness, conscientiousness, neuroticism, and openness (19). Certain personality traits are associated with depression. According to a meta-analysis of 175 studies, major depression is linked to high neuroticism and low conscientiousness and extraversion: neuroticism was a risk factor while extraversion and conscientiousness acted as protective factors (20). Another meta-analysis showed links between depressive symptoms and neuroticism, extraversion, and conscientiousness (21). This same pattern has also been reported for COVID-19 anxiety syndrome (22). These studies demonstrated that some personality traits are closely related to depression. Therefore, we hypothesized that personality traits can regulate depression.

**Figure 1:**
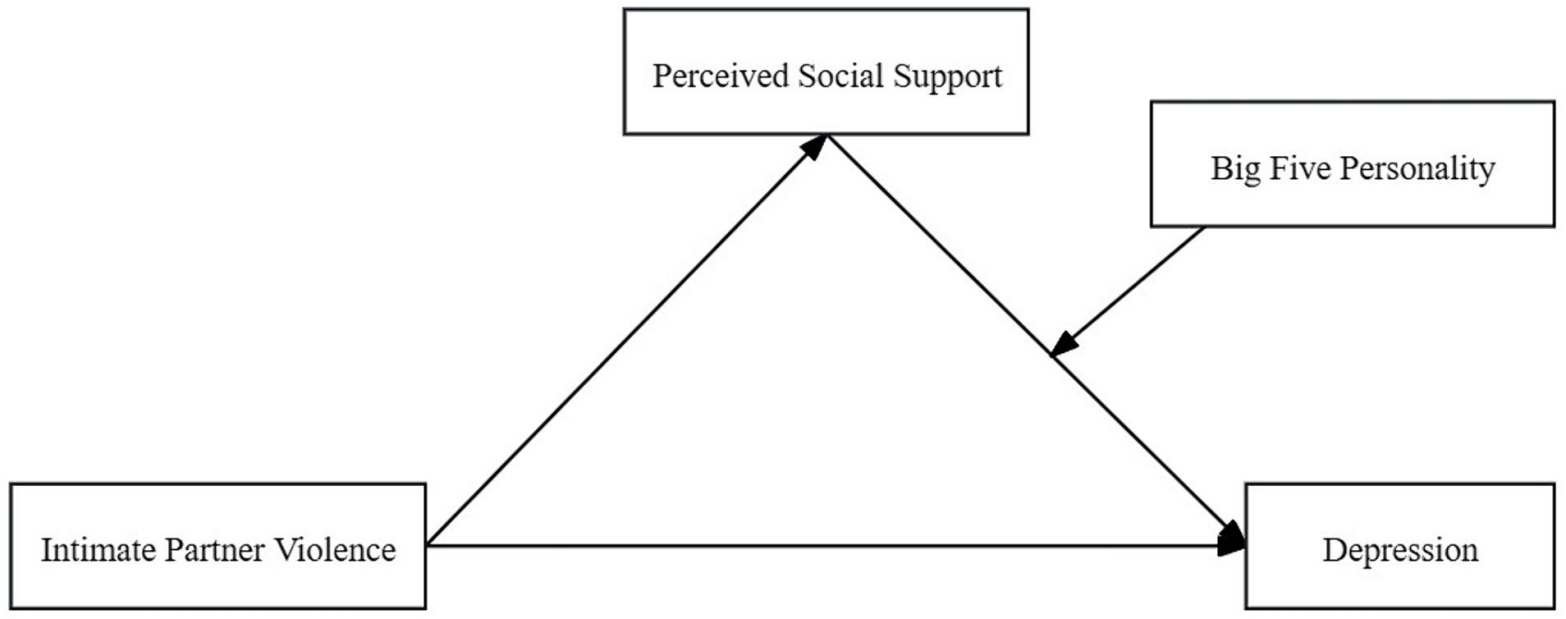
Schematic of moderated mediation model.

As shown in Figure 1, our study created a conceptual moderated mediation model to examine whether perceived social support mediates the relationship between IPV and depression and whether the Big Five personality traits moderate the relationship between perceived social support and depression.

We presented three hypotheses:

*H1:* IPV has a positive predictive effect on depression;

*H2:* Perceived social support mediates the relationship between IPV and depression;

*H3:* The Big Five personality traits moderate the relationship between perceived social support and depression.

## 2 Methods

### 2.1 Participants

The survey was conducted between June 20 and August 31, 2022, and was administered in 148 cities in the Chinese mainland, covering 23 provinces and 5 autonomous regions, including the capital cities of 4 municipalities and 2 to 6 non-provincial capitals. Data were collected through an anonymous electronic questionnaire on the WenJuanXing public online platform (https://www.wjx.cn), and 23,414 participants completed the questionnaire.

Responses were excluded if: (1) respondents completed the survey in less than 240 s; (2) the answers were logically inconsistent across items; or (3) the answers to all items were similar. Overall, 1,498 invalid responses were excluded, leaving 21,916 valid responses for further analysis. Participant ages ranged from 12 to 100 years (M = 39.43, SD = 18.85).

### 2.2 Procedure

The Institutional Ethics Committee of Shaanxi Provincial Key Research Base of Philosophy and Social Sciences-Health Culture Research Center (JKWH-2022-02) approved the research design. Before distributing the questionnaire, the investigators underwent comprehensive and specialized training that highlighted the fundamental aspects of the research, including the importance of maintaining anonymity, impartiality, and confidentiality. Participants provided informed consent before completing the questionnaire and the parents of the minor participants provided informed consent on behalf of the minor participants. The study excluded cognitively challenged participants.

### 2.3 Questionnaire

#### 2.3.1 Intimate partner violence scale

We developed the Intimate Partner Violence Scale (IPVS) by referring to Straus et al.’s (23) revised Conflict Tactics Scale, which has five items that measure aspects of three dimensions: physical, sexual, and psychological harm. Examples of items include, “My intimate partner used to directly beat or use tools to hurt me” and “My intimate partner would compare me to other people, openly insult me, and make me feel embarrassed and unconfident.” Each item was rated from 0 (*never*) to 4 (*often*); therefore, a score of 1 indicated IPV and a higher score indicated a more severe experience. Cronbach’s alpha for the present sample was 0.908.

#### 2.3.2 Patient health questionnaire

The 9-question Patient Health Questionnaire (PHQ-9), developed by Kroenke et al. (24), was used to measure depression severity in participants by surveying the participant’s psychological state over the past 2 weeks. Statements were assessed on a four-point Likert scale ranging from 0 (*not at all*) to 3 (*nearly every day*), and a higher composite score indicated more severe depression. Cronbach’s alpha for the present sample was 0.921.

#### 2.3.3 Perceived social support scale

The Perceived Social Support Scale (PSSS) was used to identify an individual’s perceived level of social support from family, friends, and significant others. Developed by Zimet (25), it is reported to have good reliability and validity in Chinese social research. Items were rated from 1 (*strongly disagree*) to 7 (*strongly agree*), with a higher overall score indicating more perceived social support. Cronbach’s alpha for the present sample was 0.880.

#### 2.3.4 Big Five inventory

The Big Five Inventory-short version (BFI-10), revised by Rammstedt and John (26), is a 10-item scale measuring the Big Five personality traits (e.g., “I see myself as someone who is reserved”). The statements were assessed on a five-point Likert scale ranging from 1 (*strongly disagree*) to 5 (*strongly agree*). The first, third, fourth, fifth, and seventh items were reverse-coded, and the sum of the corresponding question scores indicated which personality trait was most significant. Cronbach’s alpha for the present sample was 0.643.

### 2.4 Data analysis

Descriptive analyses, Pearson correlations, and common method bias tests were conducted in SPSS, version 26.0 (IBM Corp., Armonk, NY). The SPSS macro-PROCESS (model 4) was then adopted to examine the mediating role of perceived social support and the SPSS macro-PROCESS (model 14) was employed to investigate the moderating role of personality traits. To test statistical significance, 95% confidence intervals of the bias-corrected bootstrapped method were used based on 5,000 samples. Gender and age were used as control variables.

## 3 Results

### 3.1 Incidence of intimate partner violence

A total of 21,916 responses to the IPVS were included in data analysis (10,958 men and 10,958 women). Out of the 21,916 individuals surveyed, 4,454 individuals disclosed experiencing physical violence, accounting for a prevalence rate of 20.32%. Additionally, 3,869 respondents reported encountering sexual violence, resulting in a prevalence rate of 17.65%. Finally, 9,393 people reported at least one episode of psychological violence, for an incidence of 42.86%. A total of 9,709 reported experiencing IPV (determined by an IPVS score of 1 or greater), for an overall incidence of 44.30%.

### 3.2 Common method biases test

The Harman single-factor method was used to avoid the possibility of a common method bias problem. The results show that there were two factors with feature roots greater than one; the first factor explained 29.53% variation. Since it was below the 40% cut-off value, this indicated that there was no common methodological deviation.

### 3.3 Descriptive statistics and correlations

The descriptive analyses and Pearson correlation results are shown in Table 1. IPV was significantly positively correlated with depression and significantly negatively correlated with perceived social support.

**Table 1:** Results of descriptive statistics and Pearson correlation analysis for each variable.

Variable	M	SD	1	2	3	4	5	6	7	8	9
1.Age	39.43	18.85	1								
2.Violence	2.28	3.74	−0.08 **	1							
3.Perceived social support	15.03	3.78	−0.02 **	−0.24 **	1						
4.Depression	6.46	5.55	−0.10 **	0.45 **	−0.24 **	1					
5.Extraversion	6.23	1.62	−0.04 **	−0.08 **	0.19 **	−0.15 **	1				
6.Agreeableness	7.00	1.48	0.03 **	−0.19 **	0.27 **	−0.24 **	0.02 **	1			
7.Conscientiousness	6.76	1.65	0.26 **	−0.18 **	0.23 **	−0.30 **	0.19 **	0.30 **	1		
8.Neuroticism	5.73	1.56	−0.11 **	0.12 **	−0.20 **	0.31 **	−0.19 **	−0.26 **	−0.23 **	1	
9.Openness	6.46	1.55	−0.25 **	−0.01	0.10 **	−0.01	0.21 **	0.08 **	0.01 *	−0.01	1

**p < 0.01; *p < 0.05.

### 3.4 Exploring the mediating role of perceived social support

After controlling for age and gender, we used the SPSS macro-PROCESS (model 4) to examine the mediating effect of perceived social support on the link between IPV and depression (Table 2). Being subjected to violence was significantly positively correlated with experiencing depression (with a regression coefficient of *β* = 0.67, *p* < 0.05). When the mediating variable was included, IPV was significantly negatively correlated with perceived social support (*β* = −0.25, *p* < 0.05). Depression was also significantly negatively correlated with perceived social support (*β* = −0.22, *p* < 0.05), and was significantly positively correlated with being subjected to violence (*β* = 0.62, *p* < 0.05).

**Table 2:** Results for the mediating effect of perceived social support (mediation model).

Outcome variable		*R*	*R^2^*	*F*	df (1)	*p*	*β*	*t*
Depression		0.46	0.21	1950.02	3	<0.001		
	Sex						0.33	4.89**
	Age						−0.02	−10.52**
	Violence						0.67	74.72**
Perceived social support		0.25	0.06	467.19	3	<0.001		
	Sex						0.06	1.20
	Age						−0.01	−6.38**
	Violence						−0.25	−37.05**
Depression		0.48	0.23	1646.64	4	<0.001		
	Sex						0.34	5.15**
	Age						−0.02	−11.69**
	Violence						0.62	67.58**
	Perceived social support						−0.22	−24.12**

**p < 0.01; *p < 0.05; standardized regression coefficients are reported. df = degrees of freedom.

Additionally, the upper and lower bounds of the bootstrap (95% confidence interval) for the mediating effect of perceived social support did not contain zero (Table 3), indicating that perceived social support plays a mediating role in the link between IPV and depression.

**Table 3:** Mediation effect breakdown.

	*β*	Boot SE	Boot LLCI	Boot ULCI	Percentage
Total effect	0.67	0.011	0.65	0.69	
Direct effect	0.62	0.013	0.59	0.64	92.03%
Indirect effect	0.05	0.004	0.05	0.06	7.97%

Bootstrap sample size = 5,000. SE, standard error; LL, low limit; CI, confidence interval; UL, upper limit.

### 3.5 Exploring the moderating role of Big Five personality

Using gender and age as control variables, the SPSS macro-PROCESS (model 14) estimated the moderating effect of the five-factor model of personality traits within the mediation model (Table 4). Of the five traits, agreeableness, conscientiousness, and openness acted as moderators in the regression equation, while extraversion and neuroticism did not have a moderating effect in this equation; the interaction between social support and extraversion was non-statistically significant (*β* = 0.01, *p* > 0.05), as was the interaction between social support and neuroticism (*β* = 0.01, *p* > 0.05). After agreeableness (*β* = −0.18, *p* < 0.001), conscientiousness (*β* = −0.16, *p* < 0.001) and openness (*β* = −0.22, *p* < 0.001) were included as moderators in the regression equation, social support still negatively predicted depression. These findings indicate that even with moderators of agreeableness, conscientiousness, and openness, social support could still mediate the relationship between IPV and depression.

**Table 4:** Results for conditional indirect effects (moderated mediation model).

Outcome variable		*R*	*R^2^*	*F*	df (1)	*p*	*β*	*t*
Depression		0.50	0.25	1227.66	6	<0.001		
	Sex						0.38	5.78***
	Age						−0.02	−11.13***
	Violence						0.59	65.39***
	Perceived social support						−0.18	−20.10***
	Agreeableness						−0.45	−19.20***
Perceived social support × Agreeableness							−0.07	−12.49***
Depression		0.52	0.27	1319.22	6	<0.001		
	Sex						0.34	5.26***
	Age						−0.01	−3.14***
	Violence						0.59	65.31***
	Perceived social support						−0.16	−17.86***
	Conscientiousness						−0.63	−29.65***
Perceived social support × Conscientiousness							−0.04	−7.18***
Depression		0.48	0.23	1100.73	6	<0.001		
	Sex						0.34	5.17***
	Age						−0.02	−11.58***
	Violence						0.62	67.49***
	Perceived social support						−0.22	−23.91***
	Openness						−0.01	−0.53***
Perceived social support × Openness							−0.02	−3.62***
Agreeableness		Effect		Boot LLCI		Boot ULCI		
M-1SD		0.02		0.01		0.03		
M		0.05		0.04		0.05		
M + 1SD		0.07		0.06		0.08		
Conscientiousness								
M-1SD		0.02		0.02		0.03		
M		0.04		0.03		0.05		
M + 1SD		0.05		0.05		0.06		
Openness								
M-1SD		0.05		0.04		0.05		
M		0.05		0.05		0.06		
M + 1SD		0.06		0.05		0.07		

*p < 0.05; **p < 0.01; ***p < 0.001.df, degrees of freedom; SE, standard error; M, mean; SD, standard deviation; LL, low limit, CI, confidence interval; UL, upper limit.

**Figure 2:**
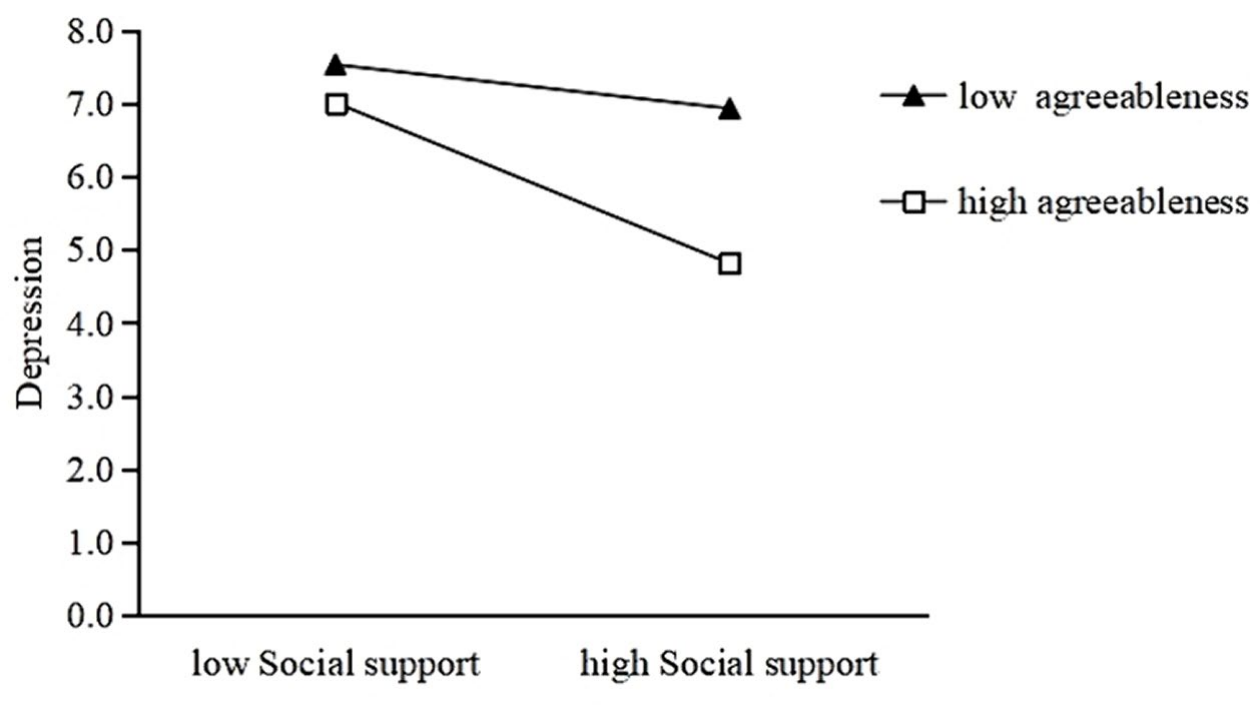
Agreeableness moderates the relationship between social support and depression.

Moreover, the interaction of social support and agreeableness (*β* = −0.07, *p* < 0.001), conscientiousness (*β* = −0.04, *p* < 0.001), and openness (*β* = −0.02, *p* < 0.001) had a significantly negative effect on depression. On this basis, three simple slope analyses were performed to deconstruct these significant interaction effects between the three traits and PHQ-9 scores. Figure 2 shows that this effect was stronger for people with high agreeableness (simple slope = −0.29, *p* < 0.001) than for people with low agreeableness (simple slope = −0.08, *p* < 0.001).

**Figure 3:**
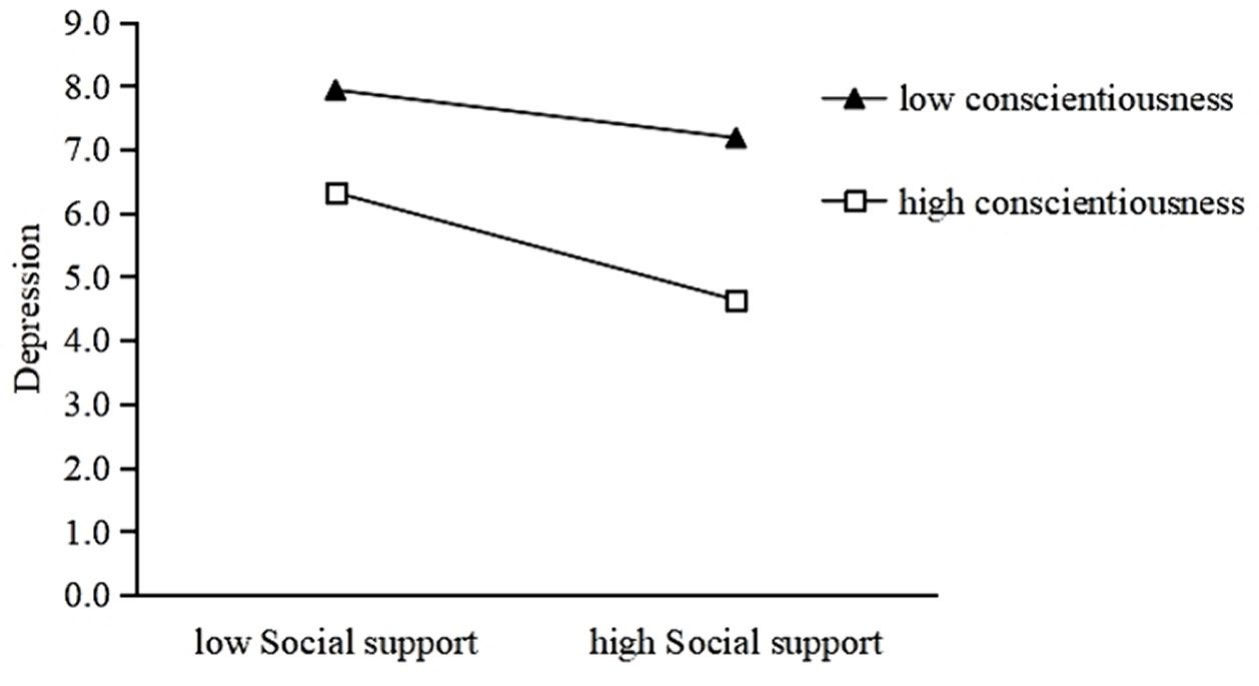
Conscientiousness moderates the relationship between social support and depression.

**Figure 4:**
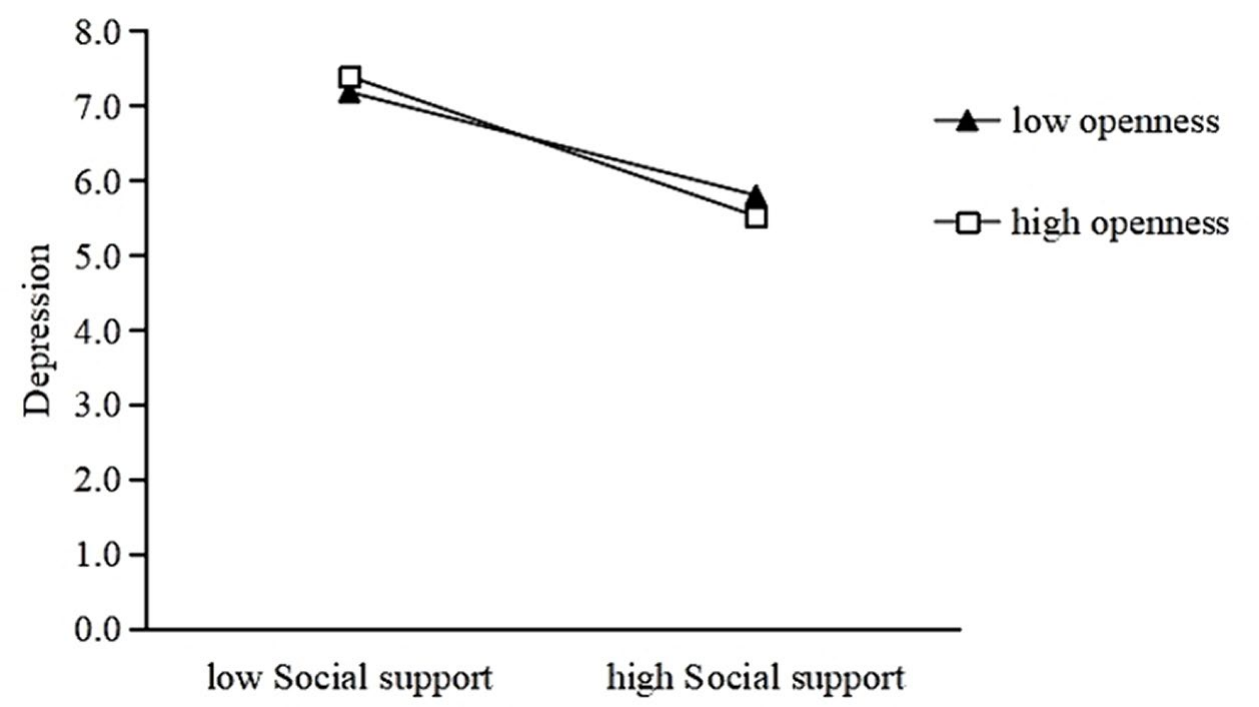
Openness moderates the relationship between social support and depression.

Figures 3, 4 show a similar effect for people with high conscientiousness (simple slope = −0.22, *p* < 0.001) and openness (simple slope = −0.25, *p* < 0.001) that was stronger than the effect in people with low conscientiousness (simple slope = −0.10, *p* < 0.001) and openness (simple slope = −0.18, *p* < 0.001). Thus, high levels of agreeableness, conscientiousness, and openness may strengthen the negative association between social support and depression.

## 4 Discussion

Based on previous research results and related theories, this study constructed a moderated mediation model to explore the relationship and mechanism between IPV and depression. We sought to answer questions about how IPV can affect depressive symptoms (mediating mechanisms) and the personality conditions under which the effects of IPV on depression were more significant (moderating mechanisms). Our findings reveal the impact of IPV on depression and provide potential direction for interventions in patients experiencing IPV.

### 4.1 Current incidence of intimate partner violence

The incidence of 44.30% in this study indicated that IPV is a serious and widespread public health problem which deserves attention. Further, participants reported a higher prevalence of psychological violence than physical or sexual violence. Psychological violence often accompanies physical and sexual violence in high proportions; however, psychological violence can exist independently (27, 28).

### 4.2 Perceived social support plays a mediating role in depression

Our results (consistent with and expanding upon previous studies) not only indicate that IPV is often accompanied by depression, but that social support could act as a mediator to reduce the negative effects of violence and symptoms of depression. Social support is an important protective factor against depression (29, 30). Nasser and Overholser (31) found that higher levels of emotional support from friends or family were significantly associated with lower levels of depression, which enhanced recovery from major depression and decreased the number of admissions to psychiatric facilities. IPV can have short- and long-term negative consequences on an individual’s physical, mental, and sexual health (32). When individuals are under stress, perceived social support can moderate their experience by enabling them to adjust their emotional stress and alleviate their depression symptoms (33). Therefore, healthcare workers should assess social support and provide adequate care or recommendations for increasing social support when patients with IPV report depressive symptoms.

### 4.3 The Big Five personality traits play a moderating role between social support and depression

The results revealed that traits of agreeableness, conscientiousness, and openness moderated the relationship between social support and depression, while extraversion and neuroticism did not. Agreeableness, conscientiousness, and openness also moderated the association between IPV and depressive symptoms; specifically, individuals with high agreeableness, conscientiousness, and openness were less likely to experience depression. Previous studies indicated links between depression and traits such as neuroticism, extraversion, and conscientiousness (34, 35). Consistent with the observations of Lewis and Cardwell (36), low conscientiousness was predictive of increased mental illness in this study, particularly depressive symptoms. Linley and Joseph (37) also found that personality traits such as extraversion, openness, agreeableness, and conscientiousness could all contribute to positive or beneficial responses after experiencing trauma and adversity. Social withdrawal and a lack of interest or engagement in activities are both manifestations of low agreeableness and openness, as well as symptoms of depression. Low conscientiousness may not cause depression directly, but it could lead to depressive experiences such as academic difficulties, job loss, and relationship problems (38). This study fills a gap between IPV and depression by revealing the personality factors that affect whether IPV leads to depression.

Our study focused on a specific sample of the Chinese mainland, which may have unique cultural factors influencing the relationship between extraversion and the variables studied. Thus, our findings are somewhat different from previous studies. In this study, the moderating effect of extraversion is non-significant on depression, whereas previous studies have shown that increased extraversion is beneficial to mental health (39). The reason may be the complexity of extraversion psychological factors. First, extraversion can be divided into two more specific aspects including Communal Extraversion and Agentic Extraversion (40). At the second level, these two aspects can be further divided into four consensual facets: Sociability, Liveliness, Venturesomeness, and Dominance (41). Communal Extraversion is negatively correlated with psychopathology, while Agentic Extraversion is often positively related to psychopathology, and too much Venturesomeness may lead to mania (42). Different features of extraversion are responsible for these positive and negative associations with psychopathology. Second, participants may have Counter dispositional behaviors, which refer to deliberately showing extraversion because they want to get better, even when this extroverted behavior is the opposite of a person’s (introverted) personality (43). Behaving discordant to one’s trait level is demanding and effortful to maintain and should therefore cause impaired levels of well-being (44).

### 4.4 Limitations

Despite the contributions of our study, we also must acknowledge this study has two limitations: First, our data are cross-sectional study which is difficult to verify the causal relationships. Second, the data were collected from self-report questionnaire measures, it is still inevitable for the information bias because of the over-reporting or under-reporting. Future studies might establish the causal relationship and mechanisms between IPV, depression, and social support by implementing longitudinal research designs. Additionally, a more objective measure of Big Five personality and depression can be used in future studies.

## 5 Conclusion

Our findings revealed that IPV is positively associated with depression and negatively associated with the mediating effect of social support. The indirect effects of IPV on depression were moderated by agreeableness, conscientiousness, and openness. The adverse effects of IPV on mental health may be mitigated by increasing available social support for patients experiencing IPV and require further research. Using these findings, patients can be coached by professionals to improve their resiliency by developing or nurturing more optimistic personality traits to bolster mental health during IPV or other setbacks, thereby helping to prevent depression.

## Data availability statement

The datasets presented in this article are available from the corresponding author, JC, upon reasonable request. Persons who have made outstanding contributions or assisted in this study may apply for the use of the data only after submitting the study hypothesis and signing a data confidentiality agreement. There is no fee for the data opening plan. Publication of the study results will include processed data only, and personal information will remain anonymous. Requests to access the datasets should be directed to JC, cjy112@i.smu.edu.cn.

## Ethics statement

The studies involving humans were approved by the Shaanxi Provincial Key Research Base of Philosophy and Social Sciences-Health Culture Research Center (JKWH-2022-02). The studies were conducted in accordance with the local legislation and institutional requirements. Written informed consent for participation in this study was provided by the participants’ legal guardians/next of kin.

## Author contributions

ZL: Writing – original draft, Writing – review & editing. YG: Writing – original draft, Data curation, Investigation, Methodology. YL: Methodology, Supervision, Writing – original draft. WX: Data curation, Investigation, Writing – original draft. LL: Investigation, Writing – original draft. SL: Investigation, Writing – original draft. HZ: Investigation, Writing – original draft. XY: Investigation, Writing – original draft. YW: Resources, Writing – review & editing. JC: Writing – review & editing.
